# Genome-wide identification of the histone acetyltransferase gene family in *Triticum aestivum*

**DOI:** 10.1186/s12864-020-07348-6

**Published:** 2021-01-11

**Authors:** Shiqi Gao, Linzhi Li, Xiaolei Han, Tingting Liu, Peng Jin, Linna Cai, Miaoze Xu, Tianye Zhang, Fan Zhang, Jianping Chen, Jian Yang, Kaili Zhong

**Affiliations:** 1 State Key Laboratory for Managing Biotic and Chemical Threats to the Quality and Safety of Agro-products, Key Laboratory of Biotechnology in Plant Protection of Ministry of Agriculture and Zhejiang Province, Institute of Plant Virology, Ningbo University, Ningbo, 315211 China; 2 Yantai Academy of Agricultural Science, Yantai, 265500 China; 3 School of Life Sciences, Yantai University, Yantai, 264005 China

**Keywords:** Histone acetyltransferases, *Triticum aestivum*, Genome-wide, Temperature, Wheat virus, Expression analysis

## Abstract

**Background:**

Histone acetylation is a ubiquitous and reversible post-translational modification in eukaryotes and prokaryotes that is co-regulated by histone acetyltransferase (HAT) and histone deacetylase (HDAC). HAT activity is important for the modification of chromatin structure in eukaryotic cells, affecting gene transcription and thereby playing a crucial regulatory role in plant development. Comprehensive analyses of *HAT* genes have been performed in *Arabidopsis thaliana*, *Oryza sativa*, barley, grapes, tomato, litchi and *Zea mays*, but comparable identification and analyses have not been conducted in wheat (*Triticum aestivum*).

**Results:**

In this study, 31 *TaHAT*s were identified and divided into six groups with conserved gene structures and motif compositions. Phylogenetic analysis was performed to predict functional similarities between *Arabidopsis thaliana*, *Oryza sativa* and *Triticum aestivum* HAT genes. The *TaHAT*s appeared to be regulated by *cis*-acting elements such as LTR and TC-rich repeats. The qRT–PCR analysis showed that the *TaHAT*s were differentially expressed in multiple tissues. The *TaHAT*s in expression also responded to temperature changes, and were all significantly upregulated after being infected by barley streak mosaic virus (BSMV), Chinese wheat mosaic virus (CWMV) and wheat yellow mosaic virus (WYMV).

**Conclusions:**

These results suggest that *TaHAT*s may have specific roles in the response to viral infection and provide a basis for further study of *TaHAT* functions in *T. aestivum* plant immunity.

**Supplementary Information:**

The online version contains supplementary material available at 10.1186/s12864-020-07348-6.

## Background

In eukaryotic cells, genomic DNA (gDNA) and histones are tightly packaged into a complex structure known as chromatin. Nucleosomes are the basic structural unit of chromatin: approximately 146 base pairs (bp) of DNA are wrapped around a histone octamer, which itself contains two molecules each of histones H2A, H2B, H3, and H4. Each histone contains a structured spherical domain and an unstructured N-terminal tail that extends from the core nucleosome [1, 2]. These tails undergo a variety of posttranslational modifications, including acetylation, methylation, phosphorylation, ubiquitination, and ADP-ribosylation. Histone acetylation is a dynamic and reversible process that is co-regulated by histone acetyltransferase (HAT) and histone deacetylase (HDAC) [3]. HAT transfers the acetyl group (CH_3_COO^−^) of acetyl-CoA to the ε-amino group (NH_3_) of specific lysine residues at the N terminus of core histones (mainly H3 and H4). Histone acetylation can neutralize the positive charge on lysine residues, weaken the binding of histones to DNA, loosen the structure of chromatin, and facilitate the binding of transcription factors or transcriptional regulatory proteins to DNA, thereby promoting gene transcription [4–7]. In general, HAT*-*mediated histone acetylation is reported to be associated with gene upregulation, but this process has been little studied in plants and requires further research [8].

Histone acetylation is important for the modification of chromatin structure in eukaryotic cells, affecting gene transcription and thereby playing a crucial regulatory role in plant development. Plant HATs are classified into four families. HACs are similar to the p300/CREB (cAMP responsive element-binding protein)-binding protein (CBP) family. HAFs are related to the TATA-binding protein-associated factor (TAFII250) family and HAMs to the MOZ, Ybf2/Sas3, Sas2, and Tip60 (MYST) family. Finally, HAGs are related to the general control non-repressible 5-related N-terminal acetyltransferase (GNAT) family with an acetyltransf_1 (AT1) domain (PF00583) and include GCN5-, ELP3-, and HAT1-like acetyltransferases [9]. As yet, *HATs* have been identified in several model plant species, including *Arabidopsis thaliana* [3], *Oryza sativa* [10], barley [11], *Vitis vinifera* [12], tomato [13], litchi [14], and *Zea mays* [15]. Silencing of *AtHAM1* and *AtHAM2* in *A. thaliana* induces severe defects in the formation of male and female gametophytes [16]. It is essential for root stem cell niche maintenance that *AtGCN5* upregulates the expression of the root stem cell transcription factors PLETHORA1 (PLT1) and PLT2 [17]. Mutations in *AtGCN5* and *AtHAF2* lead to reduced expression of light-responsive genes [18, 19]. Loss of function of *AtHAC1*, *AtHAC5*, and *AtHAC12* causes delayed flowering phenotypes [20, 21]. These findings indicate that histone acetylation plays a crucial role in the control of plant development.

Plants encounter various environmental stimuli during their life cycle, including abiotic and biotic stresses. Plant response to various environmental stresses depends largely on posttranslational nucleosome histone modifications, including histone acetylation [22]. Histone acetylation participates in the temperature regulation of plant development, and cold exposure represses the expression of four *HAT*s (*OsHAC701*, *OsHAC703*, *OsHAC704*, and *OsHAG703*) in *O. sativa* [10]. In *A. thaliana*, physical interaction of *AtGCN5* with the cold-induced transcription factor CBF1 (a C repeat/DRE binding factor) through the transcriptional coactivator ADA2b (a homolog of yeast ADA2 protein) regulates the cold accumulation process of cold-regulated (COR) gene expression [23]. Histone acetylation is also involved in the response of plants to biotic stress. In *Magnaporthe oryzae*, growth rate and spore production are significantly reduced in *MoHat1* knockout mutants, reducing their ability to infect plants [24]. A *HAT* gene (*PsGcn5*) from *Phytophthora sojae* is important for growth under conditions of oxidative stress and contributes to full virulence by suppressing host-derived reactive oxygen species [25]. SAGA (Spt-Ada-Gcn5-acetyltransferase) participates in the regulation of dicer-like2 (DCL2)-mediated transcriptional response, thereby regulating the RNAi pathway of *Cryphonectria parasitica* [26].

Although HATs have multiple roles in plant growth, development and stress response, little is known about their functions during viral infection, especially in wheat (*Triticum aestivum*). Wheat is the most widely grown crop around the globe and ranks second in importance to rice for food. However, in comparison with rice and maize, wheat is under-explored [27]. In this study, we identified and characterized members of the *HAT* gene family in *T. aestivum* and comprehensively analyzed their phylogenetic relationships, structures, chromosomal locations, expression patterns, responses to temperature stress, and responses to viral inoculation. Taken together, our results provide a set of *TaHAT* genes that have particular roles in the response to viral infection for future studies in plant immunity.

## Results

### Identification and characterization of *HAT* genes in *T. aestivum*

**Fig. 1:**
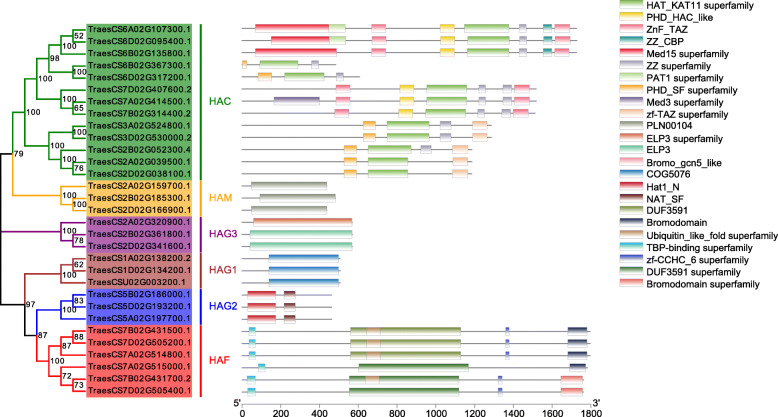
Conserved domain analysis of the TaHAT gene family. The 31 TaHATs can be divided into six groups (HAC, HAM, HAF, HAG1, HAG2, and HAG3) based on conserved domain analysis. Individual conserved domains are indicated by different colored boxes

Previous studies have shown that there are 12 *HAT*s in *A. thaliana* and eight *HAT*s in *O. sativa* [3, 10]. Here, we identified 31 *HAT*s in wheat (*T. aestivum*) by performing BLASTP searches with *A. thaliana* and *O. sativa* HAT protein sequences as queries (Table S1). HATs belong to four distinct classes: HAC, HAG, HAF, and HAM [3]. According to their conserved domains and the classification of *HAT*s in *A. thaliana*, the 31 *TaHAT*s were divided into six classes for convenient description: *HAC*, *HAG1*, *HAG2*, *HAG3*, *HAF,* and *HAM*. Each class has distinct conserved domains that support the suitability of such a grouping (Fig. 1). Details of the *TaHAT* gene family, including gene IDs, locations, and groups are provided in Table 1. Most TaHACs (85%) were 1100–1800 aa in length while *TraesCS6B02G367300.1* (484 aa) and *TraesCS6D02G317200.1* (607 aa) were special. The amino acid sequences of each class showed a high similarity. The molecular weights (MWs) of the TaHATs varied from 50.14 to 201.47 kDa. The isoelectric points (pIs) ranged from 5.23 to 8.88. *TraesCS7A02G514800.1* encoded the longest protein with the highest MW (201.47), whereas *TraesCS2A02G159700.1* and *TraesCS2D02G166900.1* encoded the shortest proteins with the lowest MWs (50.14) (Table 1). The protein properties of the TaHATs were similar to those of HATs from other plant species [3, 10].

**Table 1:** Detailed information about 31 predicted *HATs* proteins in *Triticum aestivum*

Gene ID	Location	CDS Length (bp)	Size (aa)	MW (kDa)	PI	Exons	Groups
*TraesCS2A02G039500.1*	2A:16464148–16,472,158	3558	1185	133.28	6.82	17	*HAC*
*TraesCS2B02G052300.4*	2B:25594256–25,602,539	3561	1186	133.64	7	17	*HAC*
*TraesCS2D02G038100.1*	2D:14091001–14,099,684	3558	1185	133.24	6.7	17	*HAC*
*TraesCS3A02G524800.1*	3A:739388676–739,397,596	3861	1286	144.36	7.54	17	*HAC*
*TraesCS3D02G530000.2*	3D:606995302–607,004,223	3861	1286	144.61	7.53	17	*HAC*
*TraesCS6A02G107300.1*	6A:75905141–75,917,538	5181	1726	194.15	8.62	16	*HAC*
*TraesCS6B02G135800.1*	6B:133116840–133,127,600	5181	1726	194.20	8.6	17	*HAC*
*TraesCS6B02G367300.1*	6B:641295622–641,299,743	1455	484	55.69	6.36	9	*HAC*
*TraesCS6D02G095400.1*	6D:59617005–59,629,170	5187	1728	194.26	8.64	16	*HAC*
*TraesCS6D02G317200.1*	6D:426068136–426,073,461	1824	607	70.10	6.16	10	*HAC*
*TraesCS7A02G414500.1*	7A:605759167–605,772,416	4557	1518	172.36	8.82	18	*HAC*
*TraesCS7B02G314400.2*	7B:561735305–561,748,672	4539	1512	171.76	8.88	18	*HAC*
*TraesCS7D02G407600.2*	7D:525362988–525,375,228	4557	1518	172.31	8.9	17	*HAC*
*TraesCS7A02G514800.1*	7A:700689132–700,707,173	5391	1796	201.47	5.36	21	*HAF*
*TraesCS7A02G515000.1*	7A:700795547–700,810,249	5349	1782	199.72	5.43	20	*HAF*
*TraesCS7B02G431500.1*	7B:699821564–699,839,075	5391	1796	201.46	5.38	21	*HAF*
*TraesCS7B02G431700.2*	7B:699909753–699,924,144	5289	1762	198.37	5.32	20	*HAF*
*TraesCS7D02G505200.1*	7D:610806229–610,820,455	5391	1796	201.44	5.35	21	*HAF*
*TraesCS7D02G505400.1*	7D:610841068–610,855,207	5289	1762	197.56	5.23	20	*HAF*
*TraesCS5D02G193200.1*	5D:297497242–297,502,158	1392	463	51.43	4.71	10	*HAG2*
*TraesCS5B02G186000.1*	5B:337828291–337,833,473	1392	463	51.41	4.79	11	*HAG2*
*TraesCS5A02G197700.1*	5A:401745642–401,754,640	1392	463	51.60	4.88	10	*HAG2*
*TraesCS2A02G320900.1*	2A:550539215–550,542,666	1710	569	63.57	8.88	9	*HAG3*
*TraesCS2B02G361800.1*	2B:514861001–514,865,480	1710	569	63.61	8.88	10	*HAG3*
*TraesCS2D02G341600.1*	2D:436369113–436,372,717	1710	569	63.58	8.88	9	*HAG3*
*TraesCS1A02G138200.2*	1A:230132869–230,165,911	1524	507	56.49	6.34	13	*HAG1*
*TraesCS1D02G134200.1*	1D:166822476–166,847,564	1524	507	56.47	6.25	13	*HAG1*
*TraesCSU02G003200.1*	Un:5332971–5,357,477	1524	507	56.51	6.34	13	*HAG1*
*TraesCS2A02G159700.1*	2A:107382526–107,388,480	1317	438	50.14	7.21	9	*HAM*
*TraesCS2B02G185300.1*	2B:160401169–160,407,504	1449	482	54.94	6.77	9	*HAM*
*TraesCS2D02G166900.1*	2D:110937430–110,943,241	1317	438	50.14	7.21	9	*HAM*

*CDS* coding sequence, *bp* base pair, *aa* amino acids, *MW* molecular weight, *Da* Dalton, *PI* isoelectric point

### Phylogenetic analysis of the HAT proteins

**Fig. 2:**
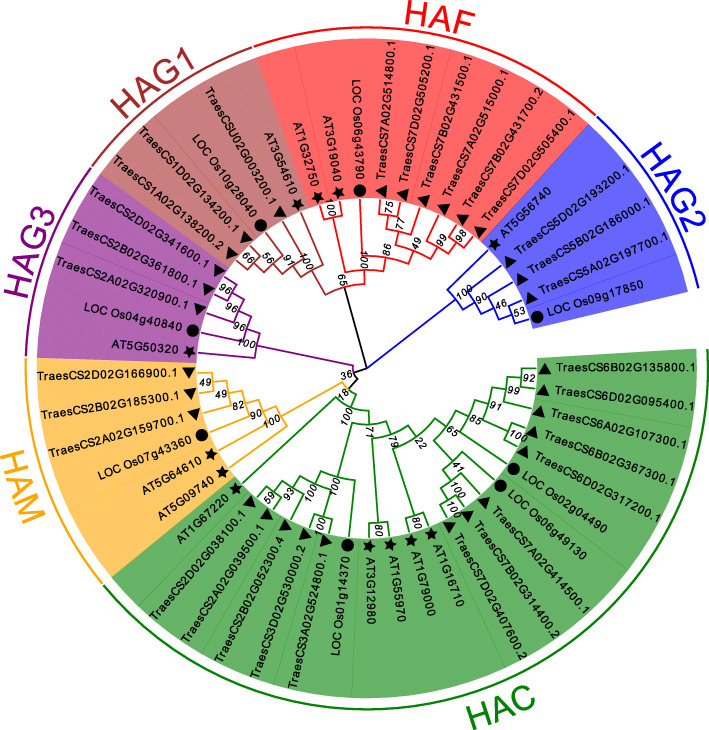
Phylogenetic tree of HAT proteins from *Arabidopsis thaliana*, *Oryza sativa* and *Triticum aestivum* constructed by the neighbor-joining method in MEGA-X. The numbers at nodes represent bootstrap values after 1000 iterations. Each group is indicated by a different color. Stars represent *A. thaliana*, circles represent *O. sativa*, and triangles represent *T. aestivum*

To analyze the phylogenetic relationships among HATs from different species, 12 *A. thaliana* (diploid), eight *O. sativa* (tetraploid), and 31 *T. aestivum* (hexaploid) HAT protein sequences were used to construct a neighbor-joining (NJ) tree. Unrooted trees that make no assumptions about ancestry illustrate only the relationships among the leaf nodes [28]. As shown in Fig. 2, HAT proteins from the three species were divided into six clades, as expected. The TaHAT proteins shared high homology with HAT proteins from other species. They clustered into the same clades with AtHATs and OsHATs with high bootstrap support values. One AtHAT, one OsHAT, and three TaHATs were clustered into groups HAG1, HAG2, and HAG3. Regardless of species, HAC was the largest group, with five AtHATs, three OsHATs, and 13 TaHATs. One AtHAT and one OsHAT were assigned to the HAM and HAF groups. The HAF group had three more TaHATs than the HAM group. These results are consistent with two previous studies of HATs from *A. thaliana* and *O. sativa*, which documented similar phylogenetic relationships among these proteins [3, 10, 28].

### Predicted structure analysis of HAT proteins

**Fig. 3:**
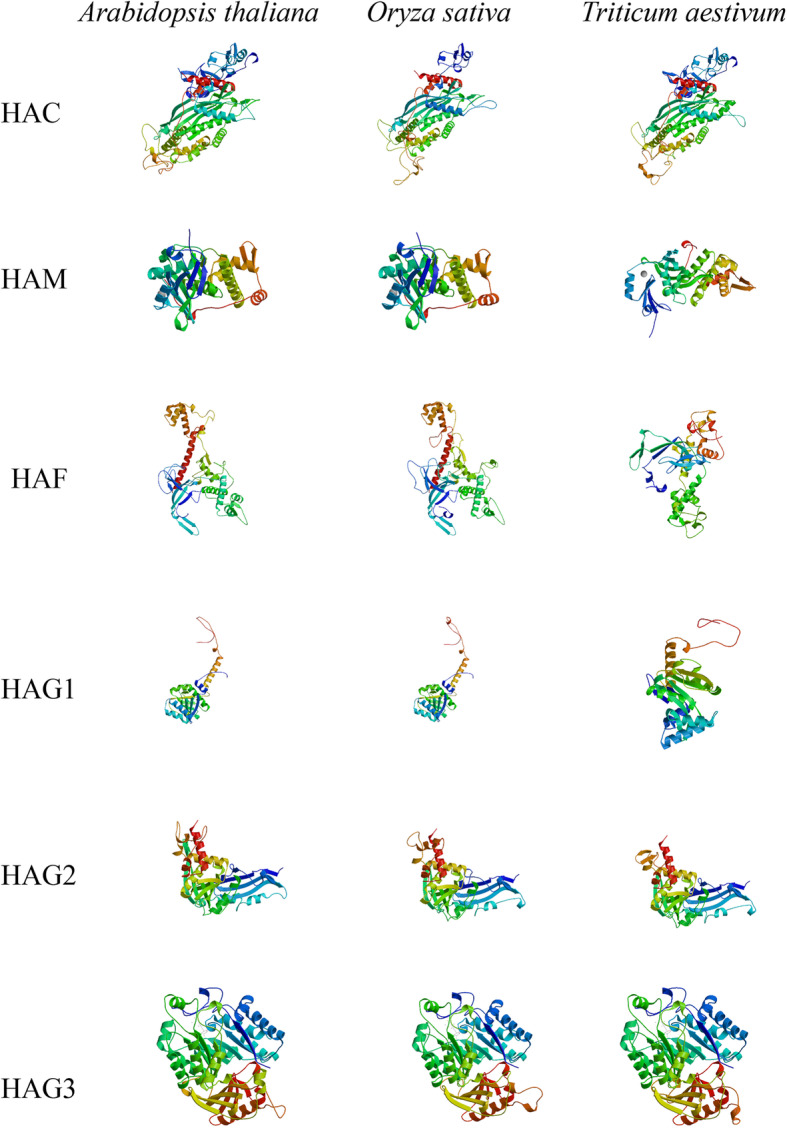
Predicted structures of *TaHATs* proteins. A gene model display is randomly selected from each group: *HAC* (*AT1G79000*, *LOC_Os06g49130*, *TraesCS6B02G135800.1*), *HAM* (*AT5G64610*, *LOC_Os07g43360*, *TraesCS2D02G166900.1*), *HAF* (*AT3G19040*, *LOC_Os06g43790*, *TraesCS7A02G515000.1*), *HAG1* (*AT3G54610*, *LOC_Os10g28040*, *TraesCS1D02G134200.1*), *HAG2* (*AT5G56740*, *LOC_Os09g17850*, *TraesCS5B02G186000.1*), *HAG3* (*AT5G50320*, *LOC_Os04g40840*, *TraesCS2D02G341600.1*)

Homology modelling has matured into an important technique in structural biology [29]. To visualize the various structures, we selected a random protein from each group in three species and modeling by SWISS-MODEL. HAC, HAG2, and HAG3 proteins had similar structures in different species. HAM, HAF, and HAG1 protein structures seemed to differ among species, but a closer look revealed that the conserved protein structures were complete and only the folding directions differed slightly (Fig. 3). As TaHACs contain two special genes which miss partial introns, we also performed protein modeling for them by SWISS-MODEL. The result showed that these two genes have similar protein structures to other family members of TaHAC (Figure S1). In general, the models of proteins from the same groups in different species were very similar, whereas those of proteins from different groups within the same species were different.

### Structures and conserved motifs of the *TaHAT*s

**Fig. 4:**
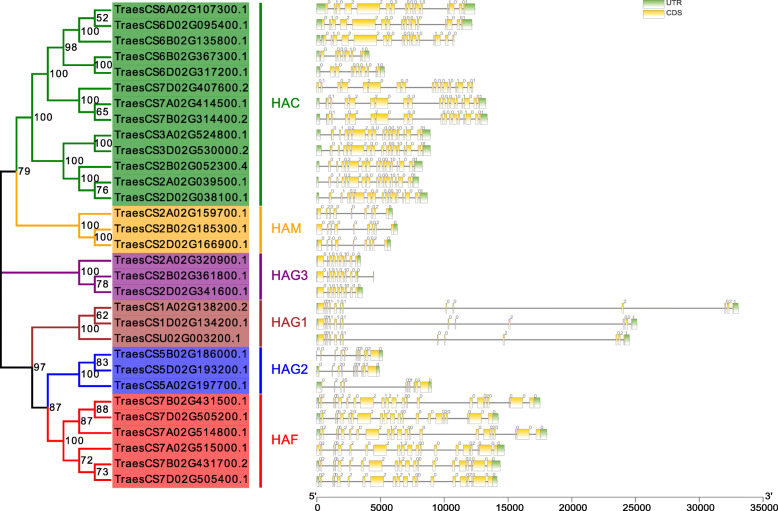
Exon–intron structures of 31 *TaHAT* genes. Exons, introns, and untranslated regions (UTR) are indicated by yellow frames, gray lines, and green frames on the right, respectively. The number on the gray line represents the number of introns

Since the comparison of gene structures provides insight into gene family evolution, we analyzed the structures of the *TaHAT* genes [30]. Analysis of gDNA sequences showed that the number of introns ranged from 2 to 14 (Fig. 4). The *TaHAT*s with highly similar gene structures were clustered together in the six main branches of the NJ tree. Most *TaHAT*s had similar numbers of introns and exons, with the exception of two *TaHAC* genes (*TraesCS6B02G367300.1* and *TraesCS6D02G317200.1*). The *TaHAF*s had the highest number of introns (14) among all the groups.

**Fig. 5:**
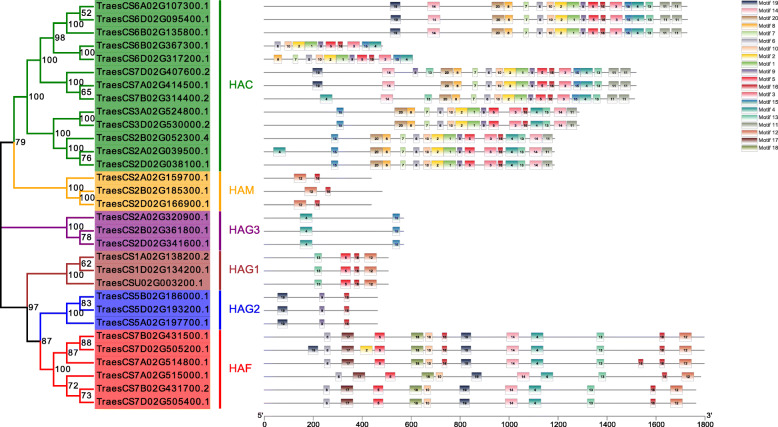
Schematic representation of twenty conserved motifs in the TaHATs. Different colored frames represent different protein motifs, and each motif has its own number

To characterize putative motifs in the wheat HAT family, the predicted amino acid sequences of the 31 TaHAT proteins were submitted to the MEME website. The result showed that 20 conserved motifs were predicted in these proteins (Fig. 5). Members of the same group contained similar motifs, suggesting that these proteins may have similar functions [31]. The HAC group had the largest number of motifs. There were probably 10 motifs in each protein, and they were arranged in the same order in the majority of sequences (motif 6, motif 10, motif 2, motif 1, motif 9, and motif 5). Motifs 1, 3, 7, 8, 11, and 20 were present only in the HAC group. Other groups also had their own unique motif sequences, and details of the 20 conserved motifs are presented in Table S2.

### Chromosomal locations and Synteny analysis of the *TaHAT*s

**Fig. 6:**
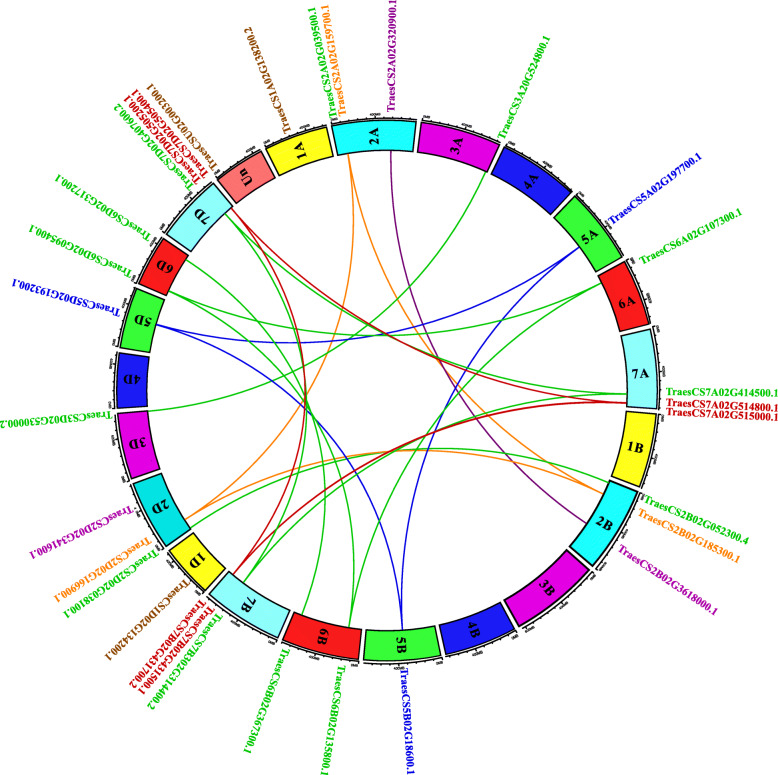
Chromosomal Locations and Synteny Analysis of *TaHAT*s. Homologous chromosomes are filled with the same color. The different colored lines indicate duplicated *TaHAT* gene pairs on different chromosomes. The positions of *TaHAT*s are marked directly on the chromosomes, and the different colored fonts represent different groups: *HAC* (green), *HAM* (orange), *HAF* (red), *HAG1* (brown), *HAG2* (blue), and *HAG3* (purple)

The *TaHAT* genes were distributed unevenly among the chromosomes of the *T. aestivum* genome (Fig. 6 and Figure S2). Three *TaHAT*s were distributed on chromosomes 2A, 2B, 2D, 7A, 7B, and 7D. Two *TaHAT*s were distributed on chromosome 6B and 6D while no *TaHAT* gene was found on chromosome 3B, 4A, 4B, 4D. Tandem and segmental gene duplications are commonly found in plant genomes [32]. Based on synteny analysis and the inspection of gene duplications, the 31 *TaHAT*s can be summarized as 12 genes (five *HAC*s, two *HAF*s, two *HAG1*s, one *HAM*, one *HAG2* and one *HAG3*), including eight genes with three copies, three genes with two copies and one gene with one copy.

### Calculation of *TaHAT* duplication events

In genetics, Ka/Ks represent the ratio between the nonsynonymous substitution rate (Ka) and the synonymous substitution rate (Ks) of two protein-coding genes. This ratio can determine whether there is selective pressure acting on the gene [33]. Collinearity and synteny analyses of chromosomes identified 21 putative paralogs in wheat (*Ta-Ta*) (Table 2) and 20 putative orthologs between wheat and rice (*Ta-Os*) (Table 3). All *Ta*–*Ta* pairs were located on homologous chromosomes (Chr2, Chr5, Chr6 and Chr7). *HAT* pairs were considered to be under purifying selection when Ka/Ks of either paralogs or orthologs were less than one. A mutation that changes a protein is less likely to differ between two species than one which is silent. Most of the time, plant eliminates deleterious mutations to avoid the protein mutation [33]. The divergence time (T) was assessed as T=Ks/(2× 9.1× 10^− 9^) million years age (Mya) based on a divergence rate of 9.1× 10^− 9^ synonymous mutations per synonymous locus per year [33]. The 21 paralogous pairs (*Ta*–*Ta*) were assessed to have diverged between 0.845 and 4.385 Mya and the 20 orthologous pairs (*Ta*–*Os*) between 23.146 and 62.318 Mya.

**Table 2:** Ks, Ka, and Ka/Ks values calculated for paralogous *HAT* gene-pairs (*T. aestivum* - *T. aestivum*)

Paralogous pairs		Ka	Ks	Ka/Ks	T (Mya)
*TraesCS2B02G052300.4*	*TraesCS2D02G038100.1*	0.0113	0.0452	0.2505	2.485
*TraesCS3A02G524800.1*	*TraesCS3D02G530000.2*	0.0108	0.0317	0.3399	1.743
*TraesCS6A02G107300.1*	*TraesCS6B02G135800.1*	0.0027	0.0277	0.0990	1.519
*TraesCS6A02G107300.1*	*TraesCS6D02G095400.1*	0.0040	0.0249	0.1601	1.365
*TraesCS6B02G135800.1*	*TraesCS6D02G095400.1*	0.0037	0.024	0.1555	1.320
*TraesCS6B02G367300.1*	*TraesCS6D02G317200.1*	0.0173	0.0445	0.3877	2.447
*TraesCS7A02G414500.1*	*TraesCS7B02G314400.2*	0.0149	0.0529	0.2812	2.906
*TraesCS7A02G414500.1*	*TraesCS7D02G407600.2*	0.0059	0.0368	0.1612	2.023
*TraesCS7B02G314400.2*	*TraesCS7D02G407600.2*	0.0146	0.0486	0.3004	2.668
*TraesCS7A02G514800.1*	*TraesCS7D02G505200.1*	0.0063	0.0587	0.1081	3.227
*TraesCS7A02G514800.1*	*TraesCS7B02G431500.1*	0.0069	0.0610	0.1140	3.349
*TraesCS7A02G515000.1*	*TraesCS7B02G431700.2*	0.0248	0.0413	0.6014	2.270
*TraesCS7B02G431500.1*	*TraesCS7D02G505200.1*	0.0092	0.0516	0.1783	2.836
*TraesCS5B02G186000.1*	*TraesCS5D02G193200.1*	0.0047	0.0154	0.3076	0.845
*TraesCS5A02G197700.1*	*TraesCS5B02G186000.1*	0.0143	0.0507	0.2814	2.783
*TraesCS5A02G197700.1*	*TraesCS5D02G193200.1*	0.0133	0.0606	0.2195	3.328
*TraesCS2A02G320900.1*	*TraesCS2B02G361800.1*	0.0016	0.0798	0.0197	4.385
*TraesCS2A02G159700.1*	*TraesCS2B02G185300.1*	0	0.0691	0	3.796
*TraesCS2A02G159700.1*	*TraesCS2D02G166900.1*	0	0.0372	0	2.042
*TraesCS2B02G185300.1*	*TraesCS2D02G166900.1*	0	0.0617	0	3.393

**Table 3:** Ks, Ka, and Ka/Ks values calculated for paralogous *HAT* gene-pairs (*T. aestivum* - *Oryza sativa*)

Orthologs pairs		Ka	Ks	Ka/Ks	T (Mya)
*TraesCS7A02G414500.1*	*LOC_Os02g04490.1*	0.2824	1.1342	0.2490	62.318
*TraesCS7A02G414500.1*	*LOC_Os06g49130.1*	0.2805	0.6306	0.4447	34.647
*TraesCS7A02G514800.1*	*LOC_Os06g43790.1*	0.1095	0.5527	0.1982	30.370
*TraesCS7B02G314400.2*	*LOC_Os02g04490.1*	0.2473	1.0127	0.2443	55.641
*TraesCS7B02G314400.2*	*LOC_Os06g49130.1*	0.2822	0.5898	0.4786	32.404
*TraesCS7B02G431500.1*	*LOC_Os06g43790.1*	0.1127	0.5477	0.2058	30.091
*TraesCS6A02G107300.1*	*LOC_Os02g04490.1*	0.0871	0.4889	0.1781	26.863
*TraesCS6A02G107300.1*	*LOC_Os06g49130.1*	0.2954	0.9333	0.3165	51.283
*TraesCS6B02G135800.1*	*LOC_Os02g04490.1*	0.0874	0.488	0.1791	26.812
*TraesCS6B02G135800.1*	*LOC_Os06g49130.1*	0.2942	0.9286	0.3168	51.023
*TraesCS7D02G407600.2*	*LOC_Os02g04490.1*	0.2737	1.0781	0.2537	59.239
*TraesCS7D02G407600.2*	*LOC_Os06g49130.1*	0.2874	0.6072	0.4734	33.362
*TraesCS7D02G505200.1*	*LOC_Os06g43790.1*	0.1108	0.5354	0.2070	29.419
*TraesCS6D02G095400.1*	*LOC_Os02g04490.1*	0.0880	0.4937	0.1782	27.128
*TraesCS6D02G095400.1*	*LOC_Os06g49130.1*	0.2948	0.9341	0.3150	51.324
*TraesCS2A02G320900.1*	*LOC_Os04g40840.1*	0.0171	0.4575	0.0374	25.138
*TraesCS2A02G159700.1*	*LOC_Os07g43360.1*	0.0226	0.4213	0.0536	23.146
*TraesCS2B02G361800.1*	*LOC_Os04g40840.1*	0.0171	0.4602	0.0372	25.284
*TraesCS2B02G185300.1*	*LOC_Os07g43360.1*	0.0226	0.4746	0.0476	26.077
*TraesCS2D02G166900.1*	*LOC_Os07g43360.1*	0.0226	0.4435	0.0560	24.367

### Expression profiles of *TaHAT*s in three-leaf-stage wheat

**Fig. 7:**
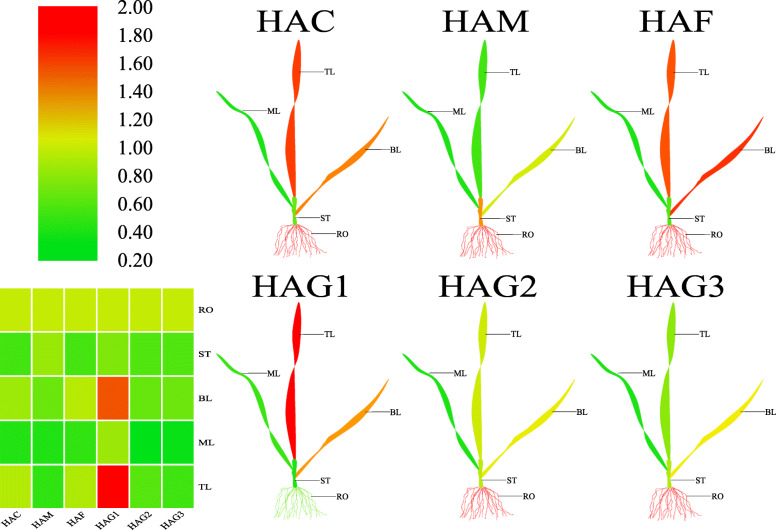
Differential expression of representative *TaHAT*s in different tissues by qRT–PCR. TL: top leaf, ML: middle leaf, BL: bottom leaf, ST: stem, and RO: roots. The mean expression value was calculated from three independent biological replicates relative to that in young leaves. The mean expression values were visualized by Tbtools; red represents a high expression level and green represents a low expression level

*HAT*s play a role in plant development [8]. To study the expression patterns of the *TaHAT* genes, one *TaHAT* from each subfamily was chosen at random for expression analysis in three-leaf-stage seedlings using quantitative real-time PCR (qRT–PCR). The plants were divided into five tissue types: top leaf, middle leaf, bottom leaf, stem, and roots. As shown in Fig. 7 and Figure S3, six *TaHAT* genes were expressed in different tissues. In addition to *HAG1*, other genes also showed high expression levels in roots. All genes showed moderate expression levels in the stem. Interestingly, gene expression levels in the bottom leaf and the top leaf were higher than those in the middle leaf. In general, faster growing wheat tissues had a higher relative expression level of *HAT* genes. These results indicate that the expression pattern of *TaHAT*s differs among tissues and is related to plant development.

### Prediction and analysis of *cis*-acting elements in the promoter regions of *TaHAT* genes

**Fig. 8:**
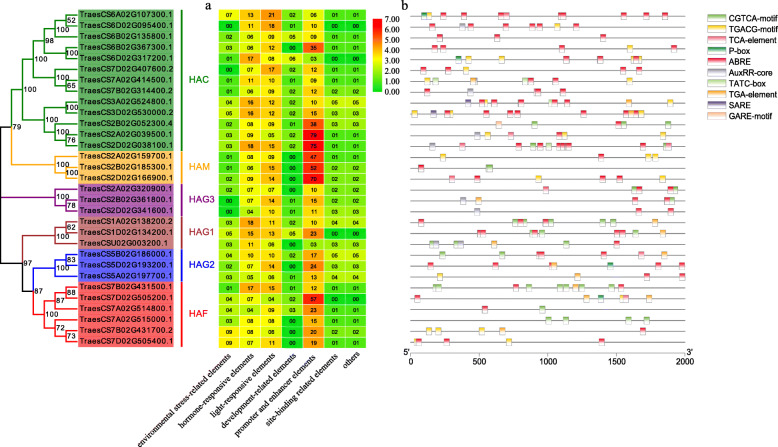
Prediction of *cis*-acting elements in the *TaHAT* promoters. **a** number of *cis*-acting elements detected in the promoter region of each *TaHAT* gene; elements were divided into seven types. **b** Type, quantity, and position of hormone-responsive elements in *TaHAT* promoters

A total of 1643 *cis*-acting elements were predicted in the promoter regions of the *TaHAT* genes. These elements associated with environmental stress, hormone response, light response, development, promoter and enhancer elements, site-binding elements, and others (Fig. 8). Hormone responsive elements were the most abundant, including auxin (IAA), gibberellin (GA), salicylic acid (SA), abscisic acid (ABA) and methyl jasmonate (MeJA). Plant growth and development are affected by various environmental stresses. Therefore, it is important to study the *cis*-acting elements associated with environmental stress [34]. In this study, 88 elements were related to environmental stress, including 25 low-temperature-responsive elements, 10 defense- and stress-responsive elements and 53 elements essential for anaerobic induction.

### Expression patterns of *TaHAT*s under different stresses

Studies have shown that the expression levels of *HAT* genes are affected by plant hormones, low temperature, drought, and salt stress [9]. To confirm that the expression of *TaHAT* genes could be regulated by abiotic and biotic stress, we tested the effects of low temperature as an abiotic stress and virus inoculation as biotic stress. The expression patterns of six *TaHAT* genes in the second leaves of 10–14 day old wheat were measured by qRT–PCR.

**Fig. 9:**
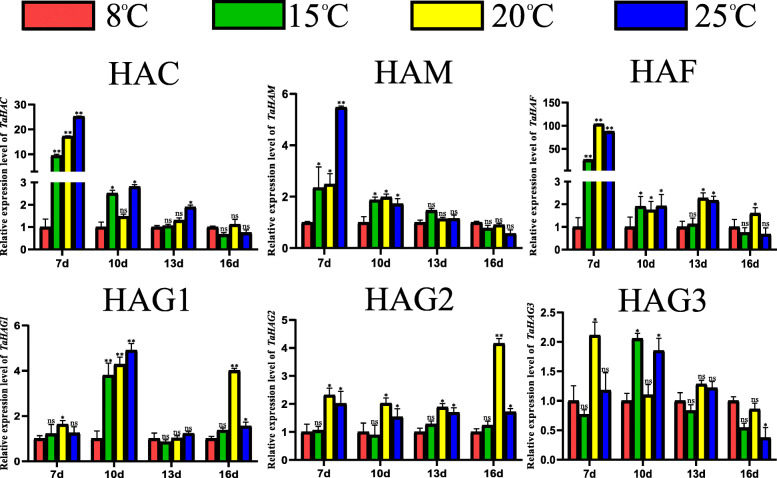
The relative expression levels of *TaHAT*s in plants grown under different temperatures for 7–16 days measured by qRT–PCR. The mean expression values were calculated from three independent biological replicates and three technical replicates. The 8 °C treatment was used as the control. (**P < 0.01; *P < 0.05; ns, *P*>0.05)

**Fig. 10:**
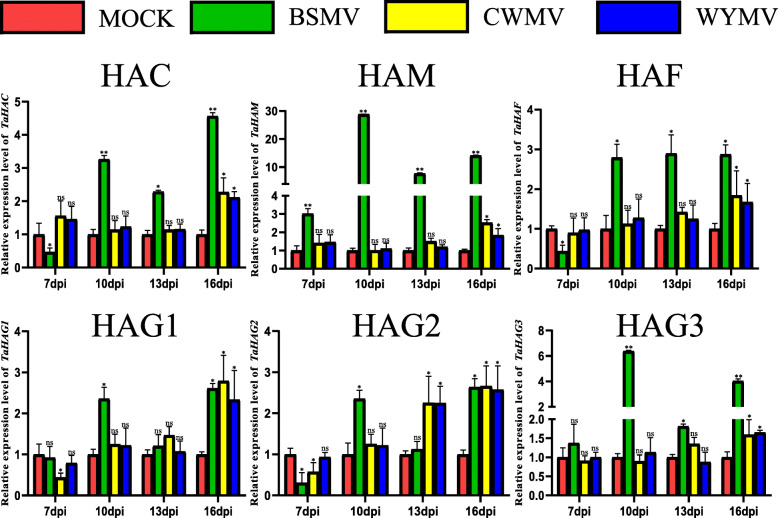
Relative expression levels of *TaHAT*s from plants inoculated with different viruses measured by qRT–PCR at 7–16 dpi. The mean (± SE) expression values were calculated from three independent biological replicates and three technical replicates. The plants inoculated with FES only (MOCK) were used as the negative controls. (**P < 0.01; *P < 0.05; ns, *P*>0.05)

The relative expression levels of *TaHAT* genes were different when wheat developed at different temperatures. Most *TaHAT* genes showed low expression at low temperatures over 7 to 10 days treatment. Among them, *TaHAC*, *TaHAF* and *TaHAG1* showed significantly lower expression level at 8 °C compared with other temperatures. As treatment time increased, there were no significant differences in the relative expression of *TaHAC*, *TaHAM* and *TaHAG3*. However, the expression level of *TaHAF*, *TaHAG1* and *TaHAG2* were still lower at 8 °C than at 20 °C (Fig. 9). In addition, *TaHAT* expression levels were upregulated at 16 days post infection (dpi) in wheat inoculated with barley streak mosaic virus (BSMV), Chinese wheat mosaic virus (CWMV), or wheat yellow mosaic virus (WYMV). The expression levels of most *TaHAT*s increased from 7 to 16 dpi (Fig. 10). Reverse transcription PCR (RT-PCR) detects whether the three viruses successfully infect wheat (Figure S4).

## Discussion

Members of the HAT family usually exist in the form of complexes and play a very important regulatory role in multiple cellular processes, including transcriptional activation, gene silencing, cell cycle regulation, DNA replication and repair, and chromosome assembly [35, 36]. HAT activity is closely related to plant growth, development, stress response, and the cell cycle process [37, 38]. *HAT* gene families have been identified and analyzed in several plants, including *A. thaliana* [3], *O. sativa* [10], barley [11], *V. vinifera* [12], tomato [13], litchi [14] and *Z. mays* [15]. In this work, we identified 31 *HAT*s in *T. aestivum* and found that their structural domains were conserved by studying phylogenetic trees, gene structures and conserved motifs. The *HAT* genes could be divided into four families: *HAC*, *HAF*, *HAM* and *HAG* [3]. We further divided the *HAG*s into *HAG1*, *HAG2* and *HAG3* based on differences in their conserved domains. After predicting their protein structures, we found that the structures of HAG1, HAG2 and HAG3 were quite different. These results indicated that they may have different functions and supporting the proposed subgroups (Figs. 1, 2, 3, 4 and 5). *TaHAT*s in each subgroup had similar gene structures and motifs, with the exceptions of the *TaHAC*s *TraesCS6B02G367300.1* and *TraesCS6D02G317200.1* (Figs. 4 and 5). However, the major domains and structures of their encoded proteins were the same as those of other *TaHAC*s (Fig. 1). We speculated that these two genes lost introns through duplication events over evolutionary time, as this is a common phenomenon [28]. On the basis of chromosome locations, we found that most genes were present in three copies, consistent with the existence of three homologous chromosomes in the wheat genome. Genes that have fewer than three copies may have experienced gene loss over the course of evolution (Fig. 6). During the evolution of *TaHAT*s, duplicated genes may have lost functions or gained new functions [39]. Notably, 31 *HAT*s were identified in the wheat genome, whereas only 12 and 8 are present in *A. thaliana* and *O. sativa*, respectively [3, 10]. This result indicates that a higher level of *HAT* gene duplication occurred during the evolution of wheat and may be attributed to wheat’s allohexaploid genome and complex evolution [40].

Although histone modifications are thought to play an important role in growth, development processes and stress responses, the specific functions of *HAT*s in *T. aestivum* remain unknown [9]. Many previous studies have shown that ortholog analysis is a feasible method for predicting the unknown functions of homologous genes from different species [41]. Since orthologs are derived from a single gene in the last common ancestor of two or more species, they often have the same function in newly evolved taxa [40]. Therefore, to predict the potential biological roles of the TaHATs, we reviewed known examples of these enzymes in *A. thaliana* and *O. sativa* to perform functional identification, and we determined the closest wheat ortholog based on phylogenetic analysis (Fig. 2). We also built protein models for each group and found that protein structures were similar within groups, demonstrating the suitability of this method for comparisons of different species (Fig. 3). In vivo, *AtHAC1*, *AtHAC5* and *AtHAC12* play redundant roles in the promotion of flowering by inhibiting the expression of FLC (FLOWERING LOCUS C) [20, 21]. Similarly, it has been reported that *AtHAC1* regulates factors upstream of FLC at flowering time through epigenetic modification and also interacts with the tomato heat stress transcription factor HsfB1 in vitro and in vivo [42]. The *TaHAC* homologs with *AtHAC*s may therefore influence flowering in wheat (Figs. 2 and 3). Since *AtHAM1* and *AtHAM2* are involved in the formation of male and female gametophytes [43], we hypothesize that the five closest *TaHAM* orthologs may perform the same function (Figs. 2 and 3). RNAi-mediated *AtHAF1* gene silencing in *A. thaliana* confers resistance to Agrobacterium-mediated transformation [44]. *AtHAF2* regulates the expression of several cold-regulated genes, regardless of its *HAT* activity [45]. Based on phylogenetic analysis and similarities in their protein models, *TaHAF*s may have similar functions to *AtHAF1* and *AtHAF2* (Figs. 2 and 3). *AtHAG1* plays a crucial role in cell differentiation and leaf and flower organ formation [46]. *TaHAG1* is highly expressed in young leaves (Fig. 7) and may share similar function with *AtHAG1* according to phylogenetic analysis and protein models results (Figs. 2 and 3). These results suggest that *TaHAG1* may have a role in plant development. Histone H4K12 is acetylated by *AtHAG2* [47], accordingly, the *AtHAG2* homolog *TaHAG2* may also perform the same function (Figs. 2 and 3). *AtHAG3* interacts with RNA Pol II in the process of transcript extension and cell proliferation during organ growth [43]. *AtHAG3* can also regulate plant response to ABA [48]. Moreover, *AtHAG3* RNAi lines are resistant to Agrobacterium-mediated transformation [10, 44]. *TaHAG3* may also have similar functions, as it clusters together with *AtHAG3* in the phylogenetic tree and has a highly similar three-dimensional structure (Figs. 2 and 3). These analyses strongly suggest that *TaHATs* play important roles in the growth and development of wheat. More detailed characterization of their functions can provide guidance for the cultivation of superior wheat varieties and for increasing wheat yield.

The relative expression levels of HATs change significantly under various biotic and abiotic stresses [9]. For example, in *O. sativa*, cold exposure represses the expression of four HATs (*OsHAC701*, *OsHAC703*, *OsHAC704*, and *OsHAG703*) [10]. The same phenomenon was observed here for all *TaHAT* genes (Fig. 9). In particular, *TaHAF* expression was upregulated 50- to 100-fold at 7 days of cold exposure. These results indicate that the expression level of *TaHATs* may be inhibited at low temperatures, thereby reducing the level of histone acetylation. However, as the treatment duration increased, the relative expression levels of *TaHAC*, *TaHAM*, and *TaHAG3* at different temperatures ceased to be significant (Fig. 9). HAG1, HAG2 and HAG3 were reported to have different functions in *Arabidopsis* [49]. HAG2 is regulated by E2F transcription factors that induce the transcription of genes required for cell cycle progression and DNA replication [50]. Here, we found HAG2 sharply increased at 20 °C at 16 d compared to those of other members. So we putative that HAG2 may participate in the regulation of some wheat genes expression at 20 °C. We speculate that these changes may reflect the acclimation of wheat to the environment. Vernalization refers to the phenomenon whereby plants must undergo a period of continuous low temperature before they can transition from vegetative to reproductive growth [51]. Further research on the relationship between vernalization and TaHATs may provide a new direction for the improvement of wheat yield. Although the function of HATs in response to cold exposure has been investigated, little is known about their potential role in response to viruses. After inoculation with BSMV, CWMV, or WYMV, the expression levels of *TaHATs* continued to increase from 7 to 16 dpi in parallel with increased virus accumulation (Fig. 10). HAC is homologous to animal p300/CREB (cAMP-responsive element-binding protein)-binding proteins, participating in many physiological processes, including proliferation, differentiation and apoptosis [52]. Previous study has reported that HACs are involved in the ethylene signaling pathway [53]. It is well known that plant hormone plays multiple roles during the interaction of plant with virus. The expression of HAC in the wheat-BSMV interaction is higher than those of others. These result indicated that HAC may play different roles in response to different viral infection. It is worth noting that many wheat viruses infect plants at low temperatures. For example, the most suitable temperature for wheat streak mosaic virus (WSMV) infection is 15 °C [54], the most suitable temperature for CWMV infection is 17 °C [55]. Virus accumulation increases at low temperatures [56]. It is also interesting that WYMV and CWMV have hidden symptoms that leaves turn green at high temperatures of about 24 °C [57]. But BSMV has no hiding symptom of high temperature. Based on the results of our study, we can speculate that decreased acetylation levels at low temperature may predispose wheat to infection by various viruses in the field, whereas increased acetylation levels at high temperature may cause the hidden symptoms of WYMV and CWMV. Of course, these remain hypotheses at present. Further research will be required to verify the relationship between TaHATs, temperature, and viral infection, thereby laying a foundation for future research directions.

## Conclusions

We identified 31 *TaHAT*s and demonstrated that they could be divided into six groups. On the whole, members of the same groups were likely to have similar functions based on their similar structures and shared conserved motifs. The expression patterns of selected *TaHAT*s differed among different tissues and at different temperatures. All of the measured *TaHAT*s were upregulated after inoculation with BSMV, CWMV, and WYMV. These results indicate that *TaHAT*s may be involved in wheat growth and development and play important roles in the response to stresses, including temperature extremes and viral infection. This work provides a basis for further functional characterization of *TaHAT*s during wheat development and for exploring the relationship between *HAT*s, temperature, and viral infection in wheat.

## Methods

### Identification of the *TaHAT* family

Previously identified AtHAT and OsHAT protein sequences were downloaded from the Ensemble Plants database (http://plants.ensembl.org/index.html) [58] in order to identify all HAT proteins in *T. aestivum* using the AtHAT and OsHAT sequences as queries. Thirty-one putative TaHATs were identified through BLASTP searches (E< 10^− 5^, %ID> 50) performed against the Ensemble database. Next, AtHAT and OsHAT sequences were submitted to the Pfam database (http://pfam.xfam.org/) and the NCBI CD-search program (https://www.ncbi.nlm.nih.gov/Structure/cdd/wrpsb.cgi) to obtain information on conserved protein domains in the HAT family [59, 60]. We used the same method to analyze conserved domains in the 31 putative TaHATs and confirm that they belonged to the HAT family, and TBtools software was used for visualization [61]. Finally, detailed information on the TaHATs, such as CDS, pI, and MW, were downloaded in batches from the Ensemble Plants database.

### Multiple alignments and phylogenetic analysis

First, a multiple sequence alignment of AtHAT, OsHAT, and TaHAT protein sequences was imported into MEGA-X and used to construct an unrooted phylogenetic tree with the neighbor-joining method and 1000 bootstrap replicates [62]. Finally, we used the online tool EVOLVIEW (https://evolgenius.info/evolview-v2/#login) to create improved graphical presentations of the trees [63].

### Structure prediction of *TaHATs* protein

We used SWISS-MODEL (https://swissmodel.expasy.org/) to make predictions for the structures of *TaHATs* protein [29]. Randomly select a gene from each group and different species for display: *HAC* (*AT1G79000*, *LOC_Os06g49130*, *TraesCS6B02G135800.1*), *HAM* (*AT5G64610*, *LOC_Os07g43360*, *TraesCS2D02G166900.1*), *HAF* (*AT3G19040*, *LOC_Os06g43790*, *TraesCS7A02G515000.1*), *HAG1* (*AT3G54610*, *LOC_Os10g28040*, *TraesCS1D02G134200.1*), *HAG2* (*AT5G56740*, *LOC_Os09g17850*, *TraesCS5B02G186000.1*), *HAG3* (*AT5G50320*, *LOC_Os04g40840*, *TraesCS2D02G341600.1*).

### Gene structures and motif analysis

The genome annotation file (GTF) for *T. aestivum* was acquired from the Ensemble Plants database (http://plants.ensembl.org/index.html) [58]. Gene structures were analyzed with TBtools Gene Structure View (Advanced) using the *T. aestivum* GTF file and the Newick Tree String of the *TaHAT*s [61]. The Multiple Em for Motif Elicitation (MEME) online tool (http://alternate.meme-suite.org/tools/meme) was used to analyze protein motifs, with a maximum selection of 20 motifs [28].

### Chromosomal locations and Synteny analysis

The initial *TaHAT* chromosomal positions and chromosome lengths were downloaded from the Ensemble Plants database (http://plants.ensembl.org/index.html), and MapChart software was used to visualize the distribution of *TaHAT*s on chromosomes [58]. To investigate duplication of the *TaHAT*s, we used TBtools to perform *TaHAT* synteny analysis [61].

### Calculation of Ka/Ks values

The Ka/Ks ratio of homologous gene pairs was used to determine whether they were under selection. *Ta*–*Ta* (*T. aestivum–T. aestivum*) gene pairs were treated as paralogs and *Ta*–*Os* (*T. aestivum–O. sativa*) pairs as orthologs. When Ka/Ks is greater than one, genes are subject to positive selection. A Ka/Ks of one implies neutral selection, and a Ka/Ks less than one indicates purifying selection. TBtools software was used to calculate Ka/Ks ratios and to estimate divergence times (T) according to T=Ks/(2× 9.1× 10^− 9^) Mya [33].

### *Cis*-acting elements in the *TaHAT* promoter regions

The 2000 bp upstream sequences of all *TaHAT* genes were extracted from the Ensemble Plants database in order to identify *cis*-elements in the putative promoter regions using PlantCARE software (http://bioinformatics.psb.ugent.be/webtools/plantcare/html/) [64]. The identified *cis*-acting elements were classified by their different functions and visualized using TBtools software [61].

### Plant materials, growth, and virus inoculation

Yangmai 158 wheat was grown in a glasshouse at 23 °C with a 16 h light, 8 h dark photoperiod. Stress treatments were applied when the wheat had reached the three-leaf stage. Three-leaf-stage wheat was then used to analyze gene expression profiles under temperature stress and viral inoculation and in five tissue types: top leaf (TL), middle leaf (ML), bottom leaf (BL), stem (ST) and roots (RO). RO tissue was treated as the control.

For temperature stress treatments, plants were placed at different temperatures (8, 15, 20, and 25 °C) in growth cabinets with 16 h light/8 h dark photoperiod. The plants placed under 8 °C were used as the controls.

For the virus inoculation treatments, plants were inoculated with barley streak mosaic virus (BSMV), Chinese wheat mosaic virus (CWMV), or wheat yellow mosaic virus (WYMV) through in vitro transcription and mechanical friction. BSMV-based gene vectors were kindly provided by Dr. Dawei Li, China [65]. CWMV-based and WYMV-based gene vectors were kindly provided by Dr. Jian Yang, China [56, 66]. The three viruses have the same inoculation method, and we will take plants inoculated with BSMV as an example. First, plasmid transcripts of BSMV RNA α, β, and γ were linearized for in vitro transcription. Second, the linearized plasmids were mixed in a molar ratio of 1:1:1 with an equal amount of excess inoculation buffer (FES) (0.06 M potassium phosphate, 0.1 M glycine, 1% bentonite, 1% sodium pyrophosphate decahydrate, 1% celite, pH 8.5) [65, 66]. Finally, the mixture was inoculated into leaves of three-leaf-stage wheat seedlings. The plants inoculated with FES only (MOCK) were used as negative controls.

### Total RNA extraction and quantitative real-time PCR analysis

Total RNA was extracted using the TRIzol reagent (Invitrogen) following the manufacturer’s instructions and stored at − 80 °C until use. The first strand cDNA was synthesized using a First Strand cDNA Synthesis Kit (Toyobo, Kita-ku, Osaka, Japan) and 1 μg total RNA per 20 μl reaction. The qRT–PCR analysis was performed using an ABI7900HT Sequence Detection System (Applied Biosystems, Foster City, CA, USA) with Hieff qPCR SYBR Green Master Mix (Yeasen, Shanghai, China). At least three biological replicates and three technical replicates were used for all qPCR analyses in this study. The *T. aestivum cell division cycle* (*CDC*) gene (accession number XM_020313450) was used as the internal reference gene to calculate relative gene expression levels using the 2^−△△C(t)^ method [66–68]. Primer sequences used in the qRT–PCR reactions are presented in Table S3.

## Supplementary Information

**Additional file 1: Table S1.** List of *HAT*s CDS and protein sequences from *Arabidopsis thaliana*, *Oryza sativa*, and *Triticum aestivum*

**Additional file 2: Table S2.** The MEME motif sequences and lengths of the *TaHAT*s

**Additional file 3: Table S3.** Specific Primers used for this article.

**Additional file 4: Figure S1.** Predicted structures of three TaHAC proteins. *TaHAC* is *TraesCS6B02G135800.1*

**Additional file 5: Figure S2.** Chromosome locations of *TaHAT* genes. Chromosomes are represented by cylinders, and homologous chromosomes are filled with the same color. The brown font represents the *HAG1* group, the green font represents the *HAC* group, the orange font represents the *HAM* group, the purple font represents the *HAG3* group, the blue font represents the *HAG2* group, and the red font represents the *HAF* group

**Additional file 6: Figure S3.** Differential expression of representative *TaHAT*s estimated by qRT–PCR (raw data) in different tissues: top leaf (TL), middle leaf (ML), bottom leaf (BL), stem (ST), and roots (RO). Mean expression values were calculated from three independent biological replicates and are expressed relative to that of roots

**Additional file 7: Figure S4.** Reverse transcription PCR (RT-PCR) detection of BSMV, CWMV and WYMV infections. Three plants were analyzed for each treatment. Total RNA from health wheat plant was used as a negative control (−). Diluted plasmid BSMV-β, CWMV RNA 2, WYMV RNA 1 were used as the positive control (+) for BSMV, CWMV and WYMV, respectively.

## Data Availability

The data included in this article and the additional files are available. The sequences of *Arabidopsis thaliana*, *Oryza sativa* and *Triticum aestivum* are available in the Ensemble Plants database (http://plants.ensembl.org/index.html).

## Abbreviations

HAT: Histone acetyltransferase
HADC: Histone deacetylase
BSMV: Barley streak mosaic virus
CWMV: Chinese wheat mosaic virus
WYMV: Wheat yellow mosaic virus
CDC: Cell division cycle
qRT-PCR: quantitative real-time PCR
WSMV: Wheat streak mosaic virus
