# Supplementary figures and images for: Genome-wide identification of the histone acetyltransferase gene family in Triticum aestivum
Source: BMC Genomics. 2021 Jan 11;22:49. doi: 10.1186/s12864-020-07348-6 (PMC7802222; doi:10.1186/s12864-020-07348-6)

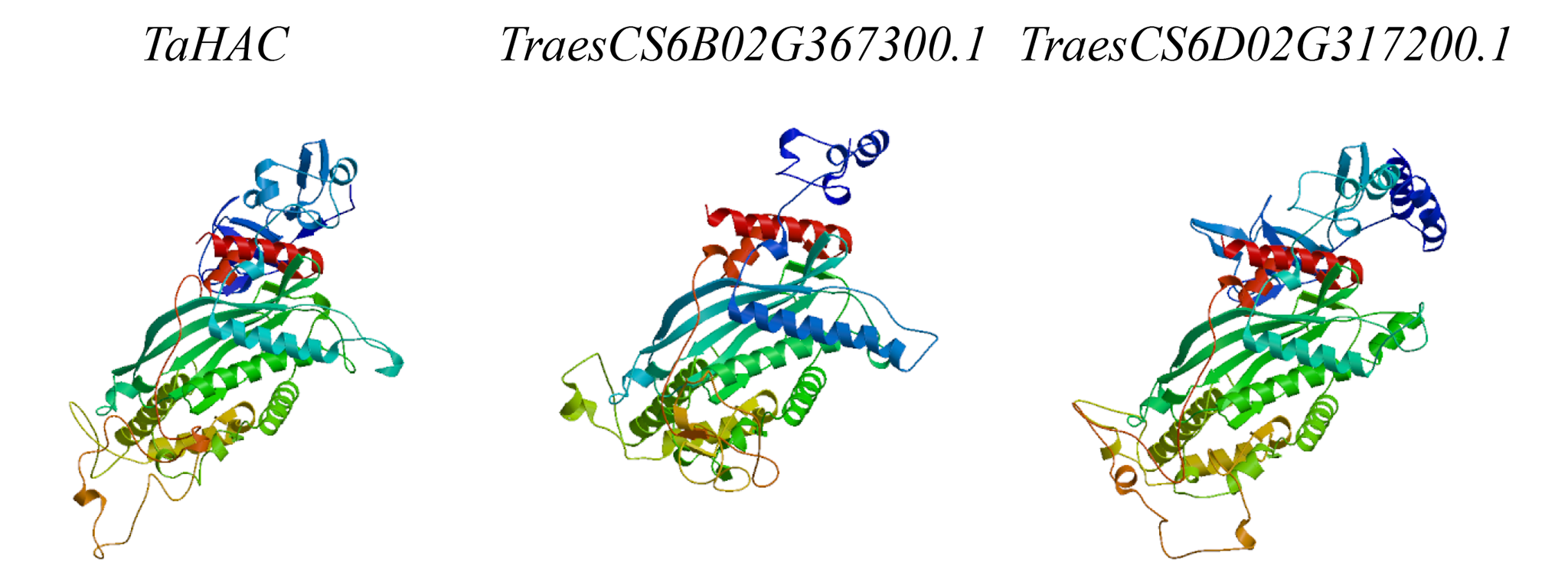

Supplement: Supplementary file 4 — Additional file 4: Figure S1. Predicted structures of three TaHAC proteins. TaHAC is TraesCS6B02G135800.1 [file 12864_2020_7348_MOESM4_ESM.tif]

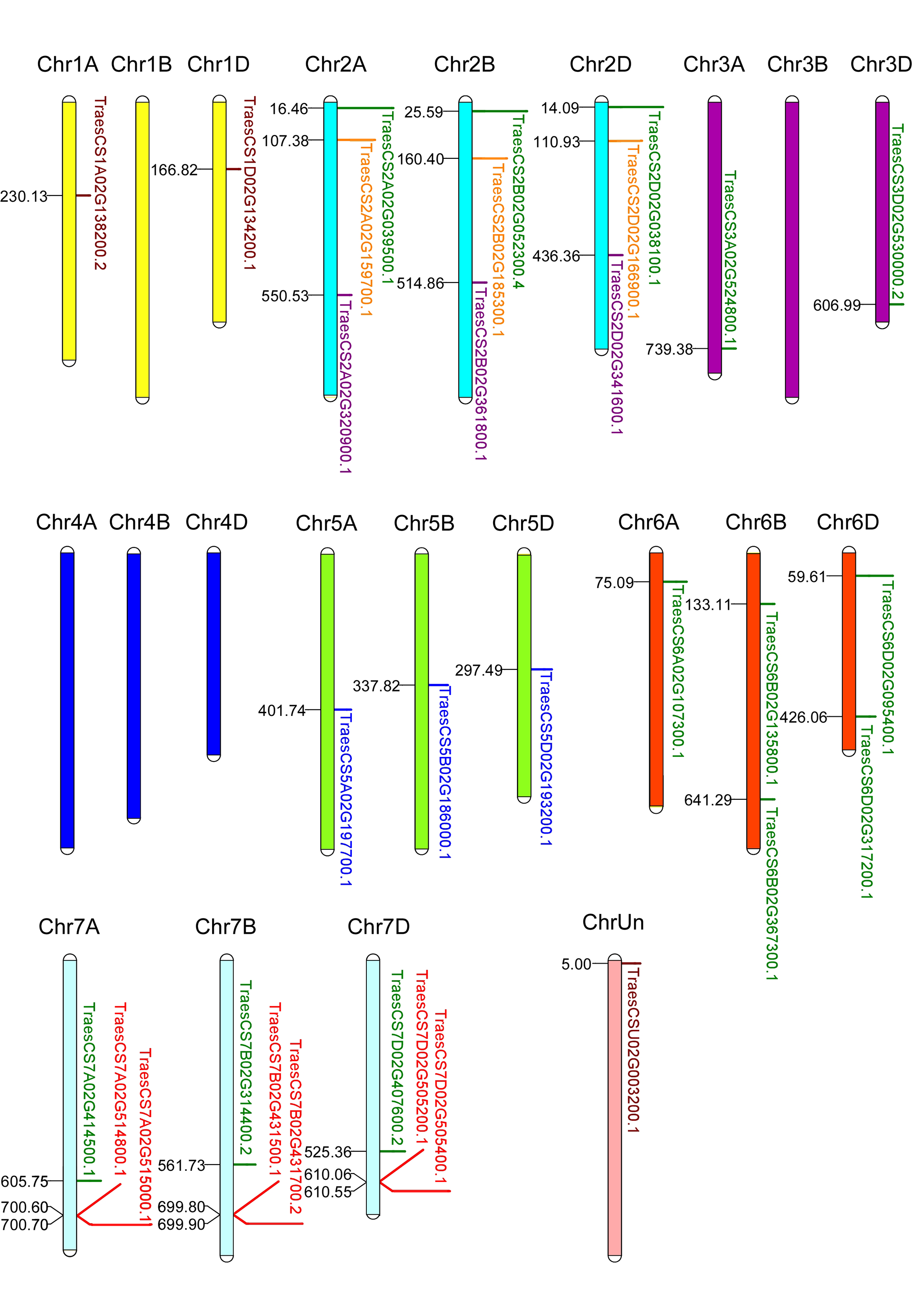

Supplement: Supplementary file 5 — Additional file 5: Figure S2. Chromosome locations of TaHAT genes. Chromosomes are represented by cylinders, and homologous chromosomes are filled with the same color. The brown font represents the HAG1 group, the green font represents the HAC group, the orange font represents the HAM group, the purple font represents the HAG3 group, the blue font represents the HAG2 group, and the red font represents the HAF group [file 12864_2020_7348_MOESM5_ESM.tif]

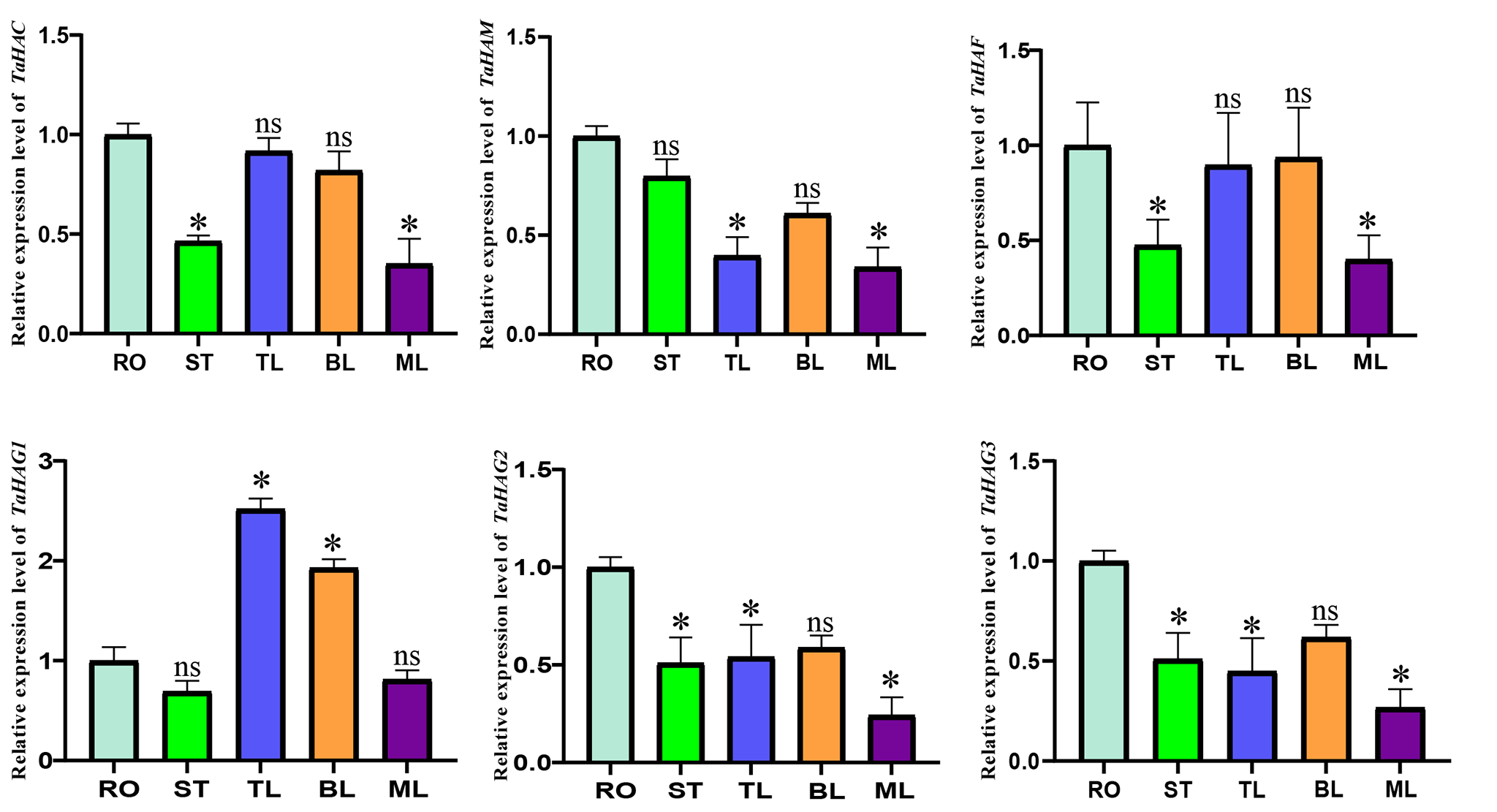

Supplement: Supplementary file 6 — Additional file 6: Figure S3. Differential expression of representative TaHATs estimated by qRT–PCR (raw data) in different tissues: top leaf (TL), middle leaf (ML), bottom leaf (BL), stem (ST), and roots (RO). Mean expression values were calculated from three independent biological replicates and are expressed relative to that of roots [file 12864_2020_7348_MOESM6_ESM.tif]

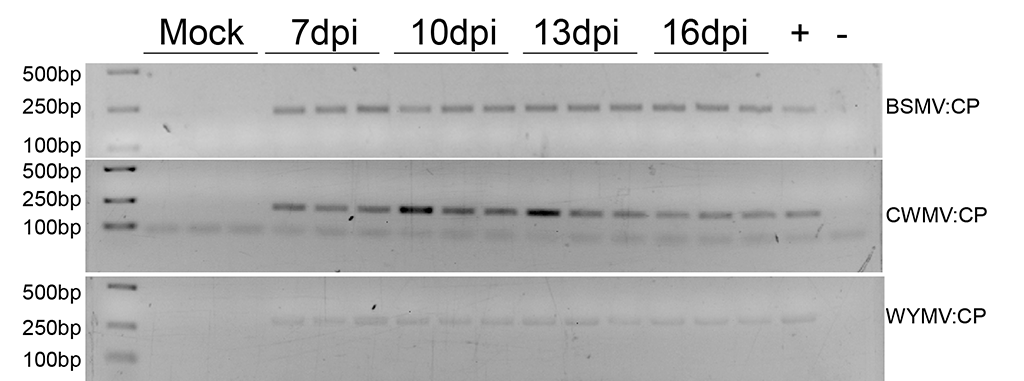

Supplement: Supplementary file 7 — Additional file 7: Figure S4. Reverse transcription PCR (RT-PCR) detection of BSMV, CWMV and WYMV infections. Three plants were analyzed for each treatment. Total RNA from health wheat plant was used as a negative control (−). Diluted plasmid BSMV-β, CWMV RNA 2, WYMV RNA 1 were used as the positive control (+) for BSMV, CWMV and WYMV, respectively. [file 12864_2020_7348_MOESM7_ESM.tif]
